# Mustardé Cheek Rotation-Advancement Flap: A Case-Based Experience in Reconstruction of a Large Defect of the Lower Eyelid Due to Squamous Cell Carcinoma

**DOI:** 10.3390/clinpract15090165

**Published:** 2025-09-15

**Authors:** Kostadin Gigov, Ivan Ginev, Petra Kavradzhieva

**Affiliations:** Section of Plastic Reconstructive and Aesthetic Surgery and Thermal Trauma, Department of Propedeutics of Surgical Diseases, Faculty of Medicine, “St. George” University Hospital Plovdiv, Medical University of Plovdiv, “Peshtersko Shausse Blvd 66”, 4002 Plovdiv, Bulgaria; kostadin.gigov@phd.mu-plovdiv.bg (K.G.); ivan.ginev@phd.mu-plovdiv.bg (I.G.)

**Keywords:** surgical flaps, mohs micrographic surgery, squamous cell carcinoma, eyelid neoplasms, reconstructive surgery

## Abstract

**Background**: Restoring the integrity of the lower eyelid presents a complex surgical challenge due to its lamellar structure and the high risk of complications. Among these, ectropion is the most frequent and troublesome outcome. **Objective**: This study aims to present a case of lower eyelid reconstruction following the excision of squamous cell carcinoma using Mohs micrographic surgery combined with the Mustardé cheek rotation flap technique, highlighting its advantages, limitations, and applicability in elderly patients. **Case presentation**: A 93-year-old female patient with right lower eyelid squamous cell carcinoma underwent Mohs micrographic surgery. The resulting defect was reconstructed using a Mustardé cheek rotation flap, chosen for its suitability in patients with adequate skin laxity. Patient-specific risk factors, including advanced age, a history of ischemic stroke, and class II heart failure (NYHA classification), were considered in the surgical planning stage. **Results**: The Mustardé cheek rotation flap provided a reliable closure with a favorable esthetic outcome and inconspicuous scarring, aligned with natural anatomical margins. The technique was technically straightforward in this patient owing to age-related skin laxity. No major postoperative complications were observed. **Conclusions**: The Mustardé cheek rotation flap represents a safe and effective reconstructive option for elderly patients with lower eyelid defects following tumor excision. This case illustrates the esthetic and functional benefits of the technique while emphasizing the need to tailor reconstruction strategies to patient comorbidities and defect characteristics.

## 1. Introduction

The eyelids play a vital role in ocular protection, lubrication, and esthetic facial symmetry. Tumors affecting the periocular region—particularly the lower eyelid—can compromise both function and appearance, making their management uniquely challenging. Squamous cell carcinoma (SCC) is the second most common malignant tumor in the facial region [1,2]. Although typically slow-growing, SCC may be locally invasive with a high expansion rate or, in other cases, highly infiltrative, often necessitating wide surgical excision with clear margins to prevent recurrence [2,3].

The lower eyelid is one of the most frequent sites of periocular SCC in elderly people due to its higher UV radiation and thinner skin. The anatomical complexity of the eyelid—comprising anterior (skin and orbicularis oculi), middle (orbital septum and fat), and posterior (tarsus and conjunctiva) lamellae—demands a reconstructive approach that addresses each anatomical structure and their functional relationships. The goals of eyelid reconstruction include restoration of lid continuity, maintenance of ocular protection, lubrication, preservation of lid mobility with no friction during bulb movement, and acceptable cosmetic outcome [1].

The study by Yamamoto et al. (2017) [4] highlights the critical role of maintaining the integrity of the lower eyelid during reconstructive procedures. The lower eyelid serves as a protective barrier for the ocular surface, and its structural and functional preservation is essential for optimal postoperative outcomes.

In their research, the authors presented a novel technique for reconstructing full-thickness eyelid defects using a combination of oral mucosa and ear cartilage strips. This approach aims to restore the inner layer (posterior lamella) of the eyelid, which is vital for maintaining the eyelid’s structural integrity. By preserving the integrity of the lower eyelid, the procedure helps prevent complications such as conjunctival or corneal irritation, which can arise from inadequate reconstruction. It also helps to minimize the risk of ectropion, which gradually leads to vision loss if not treated in a timely manner. Therefore, meticulous planning for the preservation and reconstruction of the lower eyelid is paramount in achieving successful results in oculoplastic reconstructive surgeries [4].

Multiple reconstructive options exist depending on the size, depth, and location of the defect. Small defects (<25% of eyelid length) can often be primarily closed if there is enough tissue laxity, while larger defects require more advanced reconstructive techniques, such as local flaps, composite grafts, or combined techniques. Among these, the Mustardé cheek rotation flap is a well-established technique for addressing large anterior lamellar defects of the lower eyelid, especially when direct closure or other local flaps are not possible or produce a higher risk for complications, with ectropion being the hardest one to treat [5].

This case report presents the management of a large lower eyelid defect after Mohs’ micrographic surgery of SCC with a 4 cm diameter. We describe the successful reconstruction using a Mustardé cheek rotation flap, emphasizing surgical planning, execution, and the postoperative outcome. The report aims to highlight the practical application of this technique and to discuss alternative reconstructive options in the context of periocular oncologic surgery.

## 2. Case Presentation

**Figure 1:**
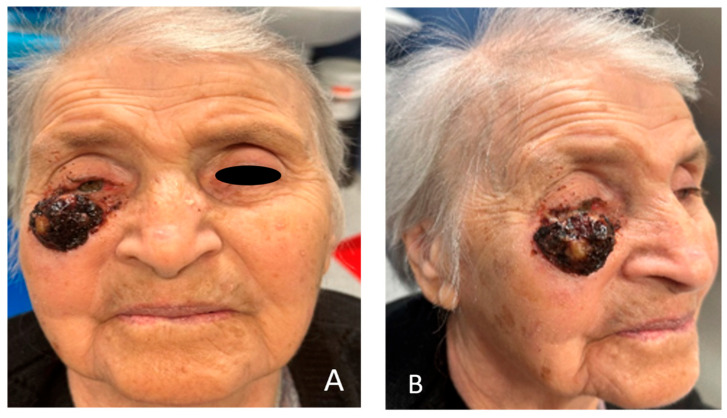
(**A**,**B**) A 93-year-old lady with an ulcerated lesion on the right lower eyelid, with a history of 10-year expansive growth and eyesight intrusion, with conjunctival hyperemia and distortion of the eyelid.

**Figure 2:**
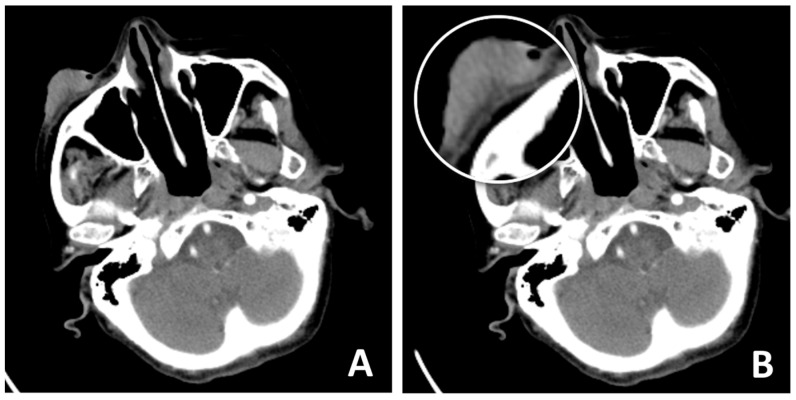
(**A**,**B**) CT scan revealing that the tumor formation engages soft tissues, to the level of the anterior lamella, with possible engagement of the orbital septum. (**B**) Underlying bone tissue appears to be intact. The lesion has more expansion rather than infiltrative characteristics.

A 93-year-old female patient presented to the clinic declaring a nearly 10-year history of expansive tumor growth in right lower eyelid without pain, and three months ago, an accelerated growth of the lesion was observed. On examination, an ulcerated lesion measuring 4 cm in diameter was observed on the right lower eyelid, extending to the malar region (Figure 1A,B). After thorough consideration of reconstructive methods, we chose to employ Mohs micrographic surgery to remove the tumor lesion and Mustarde cheek rotation-advancement flap to close the remaining defect. The patient experienced ischemic stroke 15 years ago and has a history of class II heart failure by NYHA classification, well treated with medications. After evaluation of the anesthesia risk and discontinuation of the oral anti-coagulant therapy the patient was considered eligible for elective surgery. A preoperative CT scan was performed to evaluate the involvement of the surrounding tissue (Figure 2A,B).

Mohs’ micrographic surgery is the method of choice to ensure clear margin safety and histological verification, as well as to preserve as much uninvolved tissue as possible, especially in a delicate area like the lower eyelid, which is predisposed to severe complications.

**Figure 3:**
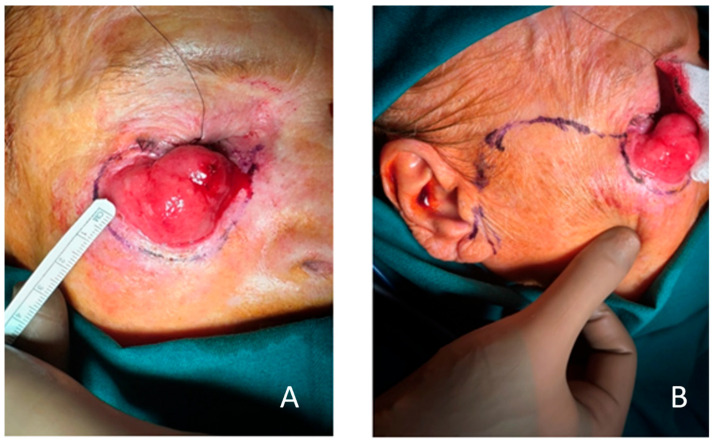
(**A**) Markings for excision with a safety margin of 4 mm. This is a critical region, and Mohs’ surgery allows us to spare tissue. (**B**) Markings of the Mustarde cheek rotational advancement flap.

**Figure 4:**
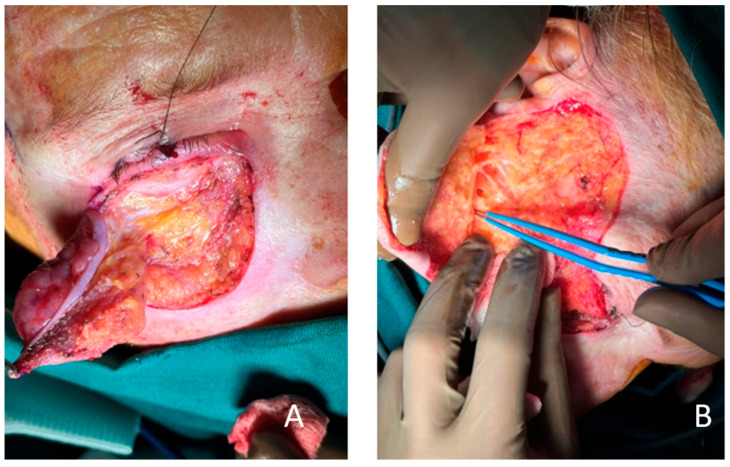
(**A**) Resection of the tumor with whole anterior lamella and partially orbital septum. Eyelid margin is spared. The area underneath the fat pads can be observed. (**B**) The cheek flap in the deep subcutaneous layer, just above the SMAS layer, is elevated with careful preservation of the branches of the facial nerve.

After markings of the borders of the lesion with a 4 mm safety margin, preserving the lid margin, and planning the Mustarde flap (Figure 3A,B), the tumor was excised along with the anterior lamella and was marked accordingly for frozen section analysis. The analysis reported squamous cell carcinoma with clear resection margins, and then we proceeded to make incisions and perform an elevation of the flap. Our incisions were continuous and starting just below the lid margin where the defect began. The incisions extended beyond the lateral canthus and reached superiorly to the temporal region. Then, we proceeded to the preauricular fold to the edge of the lobulus, and the end was at the mastoid process region. We performed the elevation and undermining of the flap beneath the subcutaneous layer, just above the SMAS layer, in order to preserve sufficient blood supply and mobility to the flap and avoid damage to the branches of the facial nerve (Figure 4B).

**Figure 5:**
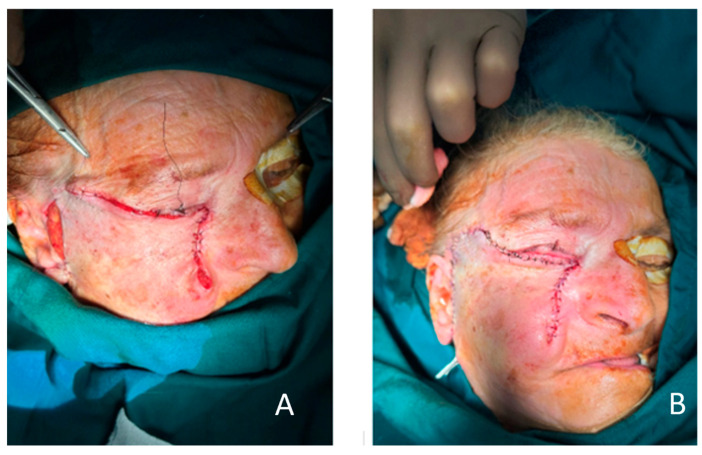
(**A**) Anchoring to the lateral and medial parts of the orbital rim and suturing of the flap with no tension were performed. (**B**) shows skin excess just beneath the eyelid margin, which we used to minimize the risk for possible future complications, such as ectropion. Immediately after surgery, eye ointment and bactigras dressing were placed.

The flap was then rotated and advanced to cover the defect. Anchoring sutures to the periosteum at the lateral and medial orbital rim were placed to secure the position of the flap and minimize risk for retraction and ectropion (Figure 5A). No tension below the lid margin was observed. The wounds were closed with 5/0 monocryl (Figure 5B).

**Figure 6:**
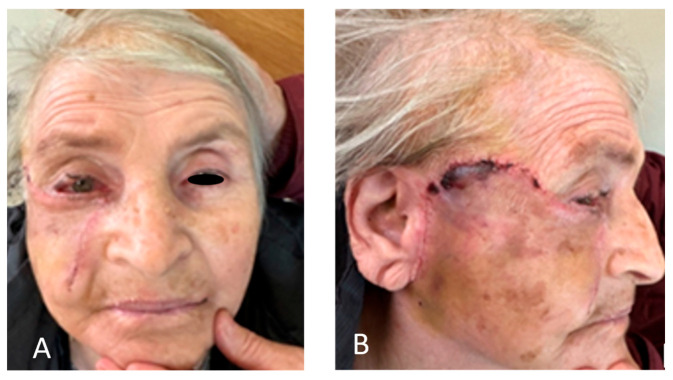
(**A**,**B**) On the 14th day, the stitches were removed. Minor swelling and slight superficial flap epidermolysis were present due to temporary ischemia.

**Figure 7:**
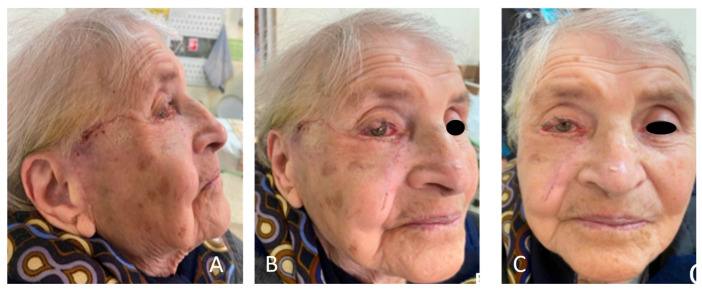
(**A**–**C**) Six weeks postoperatively, complete healing of the flap was observed with no vascular compromise. We can also evaluate the inconspicuous scarring along the lid margin and the one parallel to the nasolabial fold. The patient reported satisfactory appearance.

We observed minor swelling of the ipsilateral side of the face the next day and slight tension of the flap at its highest point in the temporal region. No infections or bleeding occurred. Postoperative care included antibiotic drops and ointments in the eyes on a daily basis, with a petroleum-based gauze over the flap. Antibiotic prophylaxis included intravenous Cefotaxime 1 g twice. After stitches were removed, steri-strips were placed at the lateral canthal region for further support for another four weeks. Additionally, in the late postoperative period and after 6 weeks, we observed a successful outcome with full recovery of the patient with improvement in the lateral canthal region. The patient refused any additional adjustments (Figure 6 and Figure 7).

Marginal flap superficial epidermolysis in the temporal area was observed as a minor complication due to temporary ischemia. However, ointment gauzes and debridement of the superficial epidermolytic layer aided in the healing process. We observed no ectropion, retraction, excessive scarring, or changes related to facial nerve damage. Moreover, the patient was completely satisfied as we managed to match the texture and color of the area with the surrounding structures using this technique. Fixed histological samples confirmed intraoperative diagnosis with clear margins and no residual tumor cells.

## 3. Discussion

Reconstruction of the lower eyelid is a complex surgical challenge that requires restoration of both functional and esthetic components. The eyelid’s role in protecting the globe and maintaining tear film integrity necessitates careful consideration in reconstructive planning.

### 3.1. Surgical Anatomy Considerations

In order to choose the appropriate technique for lower eyelid reconstruction, it is essential to evaluate the anatomical details and peculiarities of this zone. Comprehensive knowledge of eyelid anatomy is vital for surgical planning and minimizing complications in oculoplastic surgery. Structurally, the lower eyelid constitutes three lamellas. The anterior lamella is made up of thin skin and the orbicularis oculi muscle. Eyelid skin is the thinnest in the human body and is notable for its lack of subcutaneous fat, allowing it to be highly elastic. The orbicularis muscle, which controls eyelid closure, has three functional segments: pretarsal, preseptal, and orbital. Its innervation comes from branches of the facial nerve (VII). The middle lamella includes the orbital septum and the fat pads positioned posterior to the septum. The anterior surface of the septum is covered by SOOF, which is a thin fibrous layer, and this landmark is important when considering surgical intervention [6]. As an individual ages, the orbital septum gradually weakens, which leads to the protrusion of the fat pads, which can form distinctive bulging of the whole area [7]. The posterior lamella includes the tarsal plate and the palpebral conjunctiva. The tarsus is a specialized tissue made up of dense fibrous tissue mimicking cartilaginous properties, providing structure and stability. It contains fibroblasts and an extracellular matrix, along with numerous meibomian glands [8,9]. The conjunctiva, which is part of the posterior lamella, is firmly attached to the tarsal plate and helps reduce friction, supports smooth eye movement, and contributes to ocular surface health [8].

In addition to the lamellae, several structures are important to lower eyelid integrity and function and should be considered in oculoplastic reconstructive surgery. The lateral canthal tendon’s function is to firmly maintain horizontal lid position and help prevent ectropion by anchoring the eyelid laterally to the Whitnall’s tubercle on the zygomatic bone. The medial canthal tendon’s function is splitting the anterior and posterior limbs, which are located at the anterior and posterior lacrimal crests, respectively. It supports the medial portion of the eyelid. Another important structure is the capsulopalpebral fascia, which is located in the lower tarsus and assists in eyelid movement during downgaze. Lacrimal punctum and canaliculi must be taken into consideration during tumor excision and reconstruction in the medial third of the eyelid to avoid chronic epiphora [6,9].

### 3.2. Consideration of Different Techniques

Successful reconstruction relies on thorough consideration of the anatomy, diligent planning, and meticulous execution of a technique. It is important to address the defect’s size, appropriate reconstruction method, or donor site, as well as the patient’s individual peculiarities and age, as aging is characterized by prominent skin laxity, which could be critical to the choice of technique. This detailed evaluation minimizes the risk for further complications, such as eyelid retraction, excessive scarring, and ectropion with potential loss of eyesight. Also, several other principles need to be applied to achieve a good outcome: smooth contact to the bulbar surface without friction in cases where the posterior lamella is also absent, structural stability of the lower eyelids, sufficient tissue coverage for the recipient site, and minimal trauma to the donor site. Especially in cases of large lower eyelid defects, some tissue excess is required at first to avoid complications, such as retraction and ectropion, due to healing and cicatrization processes [4].

Reconstruction addressing the defect’s size is based on certain classifications, which separate sizes into several groups: when less than 25% of the eyelid surface is affected, between 25% and 50%, between 50 and 75%, and over 75% [1,3,4,5].

Techniques for lower eyelid reconstruction include direct closure, Tenzel semicircular flap, Hughes tarsoconjunctival flap, free grafts, and V-Y advancement [10,11,12,13]. Direct closure is typically utilized for defects involving less than 1/4 of the lid length and when sufficient laxity is present. A unipedicled Tripier flap or modified Tripier flap can be applied in defects involving a surface area of 1/3 to 1/2 of the lid [8,10,13]. The Tenzel flap, a semicircular skin–muscle advancement from the lateral canthal area, is suited for moderate defects (25–50%) and offers good functional and cosmetic outcomes with relatively minimal morbidity. The Hughes flap is often employed for full-thickness defects greater than 1/2 of the surface of the lower lid, particularly when posterior lamella reconstruction is required. It is a staged procedure that utilizes a tarsoconjunctival flap from the upper lid and is particularly useful when vascularized tissue is needed [8]. Free grafts, such as hard palate mucosa or auricular cartilage, may be used for posterior lamella reconstruction, typically in combination techniques. Skin grafts are a possible option when local flaps cannot be utilized [8]. However, the percentage of primary and secondary contracture increases the incidence of ectropion to nearly 15%, which is why most surgeons typically avoid this method. Moreover, there is a recognizable difference in the skin tone and texture between the graft and surrounding tissues. Also, the survival rate of the skin graft is lower than local or distant flaps [14,15].

Free microvascular flaps are another alternative to local flaps when the latter are not sufficient. A perforator anterolateral thigh flap with a fascial strip is a possible option, providing viable and adequate coverage, which is a method described by Rubino et al. [16,17].

Yamakawa et al. [18] demonstrated a novel reconstructive methodology for lower eyelid reconstruction using the transverse artery perforator flap, with the help of Doppler US, which presents several advantages, such as possible execution of the surgery under local anesthesia, decent flap mobility, and reduction in operative time. On the other hand, this flap may not be applicable in extensive lower eyelid defects, and the scarring might be too conspicuous.

Among the techniques available, the Mustardé cheek rotation flap remains a reliable and time-tested option, particularly for large lower eyelid defects involving more than 50% of the lower lid surface [19].

The Mustardé technique, used since 1964, involves a rotational flap from the cheek to resurface the anterior lamella of the lower eyelid. This flap provides excellent skin match in terms of texture and color given the proximity of donor tissue, as well as robust vascularity and a minimized risk of flap necrosis [20]. It is particularly advantageous for elderly patients or those with significant lower facial skin laxity, where cheek advancement is more easily obtained. Another great advantage of this technique is its ability to be executed in a single stage. It can also be performed in combination with canthopexy. Also, it can be combined with other techniques which address the reconstruction of the posterior lamella and provides full-thickness restoration. However, drawbacks include potential flap bulkiness, ectropion due to poor planning and insufficient recipient site coverage, distortion of the malar contour, and damage to the facial nerve with consequent facial asymmetry [21]. An alternative to the Mustardé technique is the V-Y advancement flap, which requires less dissection and undermining with less trauma, respectively. Nevertheless, the vertical vector of advancement poses a greater risk for ectropion occurrence [21,22,23].

Anastasopoulos et al. [24] reported successful treatment of lower eyelid basal cell carcinoma with tarsoconjunctival and Mustardé flaps, which address both anterior and posterior lamella defects in a combined approach. Moreover, this supports the idea that the Mustardé flap can be safely implemented in elderly patients.

In the presented case, the Mustardé flap was chosen due to the extensive nature of the defect, engaging more than 80% of the lower lid, and the need for robust vascularized tissue for coverage. Furthermore, this method allows for single-stage reconstruction, which is more appropriate for elderly patients. Mohs’ micrographic surgical method, on the other hand, allowed for maximum sparing of healthy tissue. The flap provided both coverage and contour, with satisfactory functional and esthetic results. Proper flap design, wide undermining, and tension-free closure with flap anchoring were critical in minimizing complications. In our case, tension was eliminated. Additionally, we provided tissue excess at the site of the lid margin, with initial narrowing of the palpebral fissure, in order to minimize the risk of complications after healing and cicatrization. Due to the fact that we performed extensive dissection and undermining of the flap, along with stable anchoring of the flap at the lateral and medial parts of the orbital rim, the usage of canthopexy was omitted in order to preserve the canthal tissues as much as possible if any further corrections would be needed.

Six weeks postoperatively (Figure 7A–C), we observed a successful outcome with improvement in the lateral canthal region, a lack of complications, and no tissue bulkiness or excess. The patient reported no complaints or irritation, and an ophthalmologic control check-up confirmed no corneal abrasion, dryness, or vision damage.

In conclusion, lower eyelid reconstruction requires a tailored approach depending on the size, location, and depth of the defect. While many techniques are available, the Mustardé flap remains a cornerstone for large anterior lamellar defects, offering reliable outcomes when executed with careful planning and technique.

## 4. Results

The patient’s follow-up after 6 weeks revealed a long-lasting result with restored anatomy, satisfactory appearance, and good symmetry. No complications, such as retraction, ectropion, or damage to the facial nerve, were noted. Ophthalmologic control check-up confirmed no corneal abrasion, irritation, or “dry eye syndrome”.

## 5. Conclusions

Mohs’ micrographic surgery is a preferable choice, especially when addressing large carcinomas in intricate zones, such as the lower eyelid, as we want to preserve as much unaffected tissue as possible and, at the same time, remain radical. The Mustarde technique remains a proven method for the reconstruction of large lower eyelid defects affecting over 75% of the surface. It produces durable results, abundant tissue coverage with adequate blood supply, and, if correctly executed, lower rates of complications and damage. Despite the prolonged surgery time and hospital stay, it is a single-stage procedure with a relatively low burden of care, even in patients with chronic cardiac pathology, which is treated with medications. We conclude that initial tissue excess resolves over time and aids in minimizing the risk of complications. Tissue laxity in elderly patients is a good indicator of the safe application of the Mustarde technique, especially when addressing large anterior lamellar defects. Universal techniques and algorithms for lower eyelids do not exist, and patients should be assessed individually in order to choose the most suitable treatment method.

## Data Availability

.The original contributions presented in this study are included in the article. Further inquiries can be directed to the corresponding author.

## Abbreviations

The following abbreviations are used in this manuscript:

SCC	Squamous cell carcinoma
SOOF	Suborbicularis oculi fascia
SMAS	Superficial musculoaponeurotic system
